# Ectogenesis and gender inequality: Two pathways converge

**DOI:** 10.1111/bioe.13406

**Published:** 2025-02-26

**Authors:** Jolie Zhou

**Affiliations:** ^1^ Department of History and Philosophy of Science University of Cambridge, Free School Ln Cambridge UK

**Keywords:** ectogenesis, ectogestation, gender inequality, pregnancy

## Abstract

Debate on whether ectogenesis is a morally desirable solution to gender inequality often starts by analyzing whether gender inequality has been caused by (i) reproductive differences between the sexes or (ii) social structures. I term these two sides the biological model and the social model. Without taking either side, this article contends that both models provide a fragile foundation for assessing the moral desirability of ectogenesis. I draw on Ronald Dworkin's luck egalitarian theory and Ron Amundson's perspective to demonstrate that both models are inherently interactionist and share the view that society's inadequate response to female reproductive traits is crucial in gender oppression. Actions on either biological or social factors are prima facie valid. Meanwhile, neither model can conclusively determine whether ectogenesis is morally desirable.

## 1 INTRODUCTION

The emancipatory potential of as‐yet‐unrealized human ectogenesis‐the entire gestation period occurring within an artificial womb‐has been debated in academia for some time. Some people think that gender inequality (GI) results from reproductive differences between the sexes (RDS).^1^ Others believe that it is caused by social structures (SS).^2^ These beliefs affect people's views on ectogenesis: the former see ectogenesis as a morally desirable solution to GI, while the latter are skeptical.

These opposing positions, termed the “biological model” and “social model” in this paper, suggest that if the cause of GI is primarily biological or social, the solution should align accordingly. However, it is unclear what is meant by saying that GI is biologically or socially caused. Without taking either side, I argue that both can be interpreted to suggest that the interaction between biology and society has caused GI, and neither can categorically reject or accept ectogenesis from a moral standpoint.

To examine this issue, I draw on insights from Ronald Dworkin's luck egalitarianism^3^ and the social model of disability, particularly Ron Amundson's perspectives,^4^ both of which grapple with the biology versus society dichotomy. I will show how a sensible interpretation of both frameworks can help find a way around this problem. Applying both frameworks to GI will demonstrate that there are good reasons to incorporate ectogenesis as a significant component of GI solutions, while whether ectogenesis is a morally desirable remedy remains an open question.

## 2 THE DEBATE

Feminist authors disagree on whether GI is rooted in biology or social structures, somewhat leading to their divided attitudes toward ectogenesis.

The biological model that views ectogenesis as liberating often starts with the notion that RDS have caused GI. For example, Shulamith Firestone's influential argument posits that pregnancy is the root cause of GI, necessitating the liberation of women from the “tyranny of reproduction by every means possible,” including “artificial reproduction.”^5^ A recognition of the causal influence of RDS does not require that we deny the impact of non‐RDS factors. Firestone also notes that initial biological inequality was later “consolidated and institutionalized in the interest of men” and emphasizes the importance of other radical changes, rather than viewing ectogenesis as a panacea.^6^ Other authors who endorse Firestone's position often highlight ectogenesis's potential to improve women's reproductive health, reduce gestation‐related burdens, and decouple women's social roles from pregnancy, without denying the need for social changes.^7^

What is unclear in this biological model is why the social response—consolidating and institutionalizing biological inequality to advantage men—cannot be considered a cause. The distinction between the biological cause and the later social response seems to be merely a matter of temporal sequence. Suppose a person falls and passersby do not help her, instead trampling over her and causing serious injury. Although her fall is the origin of the accident, there seems to be no compelling reason to exclude the trampling as a cause of the injury. Theoretically, cutting the later part of the causal chain—correcting the social response—could also work. The argument that biology is the origin of inequality itself cannot generate the normative guidance that eliminating this origin is the only or best path to equality.

Conversely, authors viewing GI as primarily social tend to dismiss ectogenesis as crucial for achieving gender equality. Some strongly assert that the root of GI is social, not medical, or biological.^8^ Giulia Cavaliere concedes that even if RDS have impacted GI, social mediation is the primary cause; thus, ectogenesis, which misleadingly attributes GI to RDS, should only be used to provoke attention toward unjust SS or as a minor part of a much broader political, social, and ethical agenda.^9^

Cavaliere uses an analogy to illustrate her stance: using Whitening™ to change skin color to white to end racism is morally undesirable, “regardless of its practical efficacy,” for two reasons: (1) oppressive social norms need changing, not biological traits, and (2) it promotes liberation through contentious assimilation.^10^ This analogy appears inaccurate at first glance. Pregnancy involves embodied harm and health risks for women, while skin color does not; ectogenesis bypasses biology rather than changing it. Creating “sameness” through shared means is not necessarily assimilation; for example, ramps that accommodate both wheelchair users and others are not considered such tools. Or imagine a wearable limb—Bypassing™—that facilitates effortless walking for people with mobility issues and even allows non‐impaired individuals to walk faster. Intuitively, proposing Bypassing™ to address the disadvantages faced by people with impairments seems less morally objectionable. Therefore, I will draw on disability discourse to clarify my points about the social model later.

Cavaliere's doubt about ectogenesis's emancipatory potential hinges on her assessment of GI's root and its normative role in guiding solutions. She asserts that GI is primarily social—because of this, social norms should be changed first, and because of this, ectogenesis symbolizes superficial alignment with the dominant group by “obliterating their difference”^11^ rather than accommodating diversity and addressing deeper societal structures. This stance leaves key questions unresolved:

First, is there a convincing method to weigh whether biological or social factors are primary in forming GI? Cavaliere's claim that RDS are socialized into oppressive structures does not confirm the dominance of social factors; it just presents an interaction. She also forecasts that ectogenesis will struggle to dismantle oppressive norms,^12^ but this still cannot serve as an argument for “GI is primarily social,” even if likely, since social efforts such as those in education and employment have not achieved that either.

Second and very importantly, does defending ectogenesis for gender equality necessarily mean locating GI in pregnancy? Relatedly, must accommodating biological diversity mean maintaining original biological modes? Ectogenesis is a human artifact that connects human biology with the external environment. Why can it not indicate that GI stems from this interaction?

Third, how can we determine that “social” means are *morally* superior to “biological” means? I endorse Cavaliere's distinction between practical efficacy and moral desirability. She correctly argues that biological solutions, while effective, may lack moral justification. Nevertheless, the same applies to social means—they can be effective yet morally questionable. For instance, lower fertility rates often correlate with higher gender equality.^13^ The seemingly most effective solution might be lowering gender equality, such as reverting to forced marriages and pregnancies, but this would clearly be morally unacceptable.

Finally, how can we justify medical support for women's reproductive health as crucial to gender equality? If the social model suggests that altering biological traits for equality is misleading, then medical interventions to improve reproductive safety seem even less desirable than ectogenesis, which bypasses biology. However, if social model holders aim for “a world where pregnancy does not disadvantage women socially, *physically*, or economically,”^14^ medical intervention becomes essential. It would be inconsistent to deem these interventions crucial while dismissing ectogenesis. With these points in mind, I will explore a more reasonable social model of GI in the fourth section.

In general, the simplified biological and social models face similar issues: both assume that there is a way to weigh the causal contributions of social and biological factors to GI, which I contend is arbitrary. Moreover, both consider locating the cause of GI in biology or society as a foundation for accepting or rejecting ectogenesis, but this foundation is fragile—neither can straightforwardly achieve the intended objective, nor can they categorize ectogenesis as pure “biological” or “social.” To clarify, in what follows, I will explore two frameworks that ostensibly represent “biological” and “social” causes of inequality.

## 3 THE BIOLOGICAL MODEL AND LUCK EGALITARIANISM

Ronald Dworkin's luck egalitarian theory can be seen as a kind of biological model, viewing certain inequalities as resulting from unchosen natural endowments—brute luck.^15^ It emphasizes society's moral responsibility to compensate for these inequalities, rather than the individual bearing the misfortune; accordingly, if society fails to do so, it creates systematic and structural inequality in a social sense.

Within this framework, RDS, when seen as the cause of GI, can be interpreted as brute luck. Smajdor has applied this perspective to defend ectogenesis.^16^ By further analyzing her work, I argue that interpreting GI through Dworkin's framework leaves the question of whether ectogenesis is morally desirable unresolved.

### 3.1 Dworkin's framework

Dworkin's theory of distributive justice is illustrated through a fictional scenario where shipwreck survivors allocate island resources equally, following three key steps:

1. Initial equality auction: Each survivor receives an equal number of clamshells to bid on preferred resources. Initial equality is achieved when no one envies the possessions of others.
2. Establishing *prima facie* right to compensation: Over time, initial equality is disrupted by two types of luck: “option luck,” arising from calculated risks such as planting a risky crop, and “brute luck,” involving uncontrollable risks like bad weather or illness. Compensation is warranted only for disadvantages caused by brute luck, not option luck.
3. Generating the *actual* right to compensation: With limited resources, survivors must prioritize which instances of brute luck to compensate. Standing behind a veil of ignorance, individuals can select insurance against specific types of brute luck, aware of the suffering that they cause but uncertain whether they will experience them. This process *democratically* establishes the actual right to compensation.

### 3.2 Ectogenesis as a form of compensation

The second and third stages of Dworkin's philosophical story are pertinent to GI. However, they only provide principles for identifying conditions that can generate prima facie and actual rights to compensation. Ultimately, we need to ask whether ectogenesis is a plausible *form* of compensation in this framework. Accordingly, three sequential questions are critical:

1. Can GI be seen as the result of brute luck, thereby justifying prima facie right to compensation?
2. If so, should it be prioritized to establish the actual right to compensation?
3. And, can ectogenesis be a plausible and significant form of compensation?

As previously discussed, authors viewing ectogenesis as liberating usually hold that the *origin* of GI lies in RDS. According to Smajdor, this origin can be seen as brute luck or natural inequality—stemming from the unequal biological choices available to men and women to avoid gestational harms and risks, justifying prima facie right to compensation.^17^ To establish an actual right, Smajdor suggests a proactive role for ethicists and policymakers, because “people need to be persuaded.”^18^ I believe that ethicists may function like insurance brokers in Dworkin's hypothetical scenario, encouraging individuals to purchase insurance against gestational risks. Smajdor argues that if individuals consider that about 50% of the population are susceptible to harmful burdens involved in female gestation, choosing ectogenesis as compensation becomes plausible.^19^ She also contends that such natural inequality cannot be separated from the further disadvantages that women endure.^20^ Therefore, if ectogenesis is considered a plausible solution to natural inequality, it also represents an important step toward addressing GI in her reasoning.

I agree with Smajdor that in Dworkin's framework, the natural inequality involved in reproduction entails compensating women. Yet, Smajdor's analysis seems somewhat hasty, lacking a full distinction between the right to compensation and the right to a specific form of it. Gestational harm and risks may justify prioritizing the protection of women from these issues, but they do not directly justify ectogenesis.

Dworkin's framework only identifies which inequalities caused by brute luck should be prioritized in compensation, without specifying whether the compensation should focus on biological or social aspects. Some of Dworkin's discussions suggest that compensation might take the form of finance. For instance, he illustrates, “one would count monetary compensation for the loss of his sight as worthless in the face of such a tragedy, while the other, more practical, would fix his mind on the aids and special training that such money might buy.”^21^ However, money can also serve as initial compensation to fund various pro‐equality projects, supporting expansive solutions as long as they achieve the goal. As Buchanan et al.^22^ note, luck egalitarians tend to focus on changing social factors to address natural inequalities, but their theories also inherently support using medical means to directly mitigate natural disadvantages. Accordingly, addressing inequalities resulting from RDS can involve both medical and social means.

Perhaps we can extend the democratic procedure to determine forms of compensation. After deciding which types of brute luck to insure against, people can return to the veil of ignorance to select the form of compensation. To do so, they need to be fully informed about the strengths and weaknesses of each compensatory option. There are, of course, good reasons to choose ectogenesis such as its best effectiveness in removing gestational harm and risks.^23^ However, the democratic nature dictates that the desirability of ectogenesis ultimately depends on *people's actual choices*. Smajdor also proposes an open choice for people to decide: If individuals did not know their gender at birth, would they prefer a society where women bear all pregnancy burdens and risks (Society A) or one where ectogenesis is perfected (Society B)?^24^

Perhaps the truly difficult choices lie between (i) Society A, where pregnancy is easy and safe without disadvantaging women, and (ii) Society B, where optional and perfect ectogenesis exists. Apart from considering the feasibility of the two paths, attitudes and values toward pregnancy inevitably play a crucial role in luck egalitarianism because of its democratic nature. If a society's majority prefers to accept a certain degree of gestational risk while pursuing non‐ectogenesis forms of compensation that enable women to achieve life goals beyond gestation, they may not choose ectogenesis. Therefore, the narrative that “GI is rooted in pregnancy” offers ectogenesis only an initial defense, not a conclusive one.

Another natural inequality is men's inability to experience pregnancy, which Smajdor identifies as a deficit warranting prima facie restitution.^25^ Individuals behind the veil of ignorance need to consider if ectogenesis best compensates for such a deficit if they prioritize avoiding it. At least, compared to the patriarchal approach of sacrificing women's autonomy and other life goals (discussed further in the next section), ectogenesis is morally preferable, fulfilling men's procreative desires while alleviating women's gestational burden.

It is precisely society's mishandling of the “compensation” for both natural inequalities that has led to structural gender inequality. Here, a reasonable biological model begins to converge with a reasonable social model, as I will demonstrate in what follows.

## 4 ECTOGENESIS IN THE SOCIAL MODEL OF INEQUALITY

As discussed, skeptics argue that GI is best addressed through social methods, but their reasoning is unclear on why social factors are primary or ectogenesis is “biological”; they also inconsistently support medical care for pregnancy while dismissing ectogenesis.

The disability discourse offers rich insights into similar issues. Debate on disability‐based inequality is often divided into the medical and social models. The social model, critical of the medical model, attributes inequalities to societal influences. However, if the social model only focuses on eradicating biases, discrimination, and stereotypes, it may not benefit individuals with impairments because some impairments inherently cause discomfort and limitations.^26^ They often require external facilities, technological devices, and medical interventions. Thus, a sensible social model should comprehensively address actual needs. Ron Amundson's insights, especially on tool use, can inform such a model.

### 4.1 Interaction, technology, and medical interventions

Amundson, a defender of the social model, rejects labeling traits as “normal” or “abnormal,” with the latter often perceived as less functionally capable. He contends that people just have “typical” and “atypical” traits without a value hierarchy, and those with atypical traits can function well with proper social arrangement, such as tools. For instance, people with mobility impairments can use wheelchairs and ramps to move effectively. Thus, society should offer adequate technological support for those with atypical traits.^27^

One might argue that the ability of atypical individuals to function well with technological aid does not directly justify a societal obligation to provide such support. For instance, the fact that someone can fly with the right equipment does not imply that society must provide it. I agree that moving from “can” to “ought” requires further justification.

Though not explicitly stated, Amundson and other philosophers indeed suggest a plausible reason—*fairness*. It is not just atypical individuals who need external tools; using tools for physiological functions is routine in everyday human life.^28^ Human survival depends on interacting with the environment, and humans excel at niche construction.^29^ As Tim Lewens elaborates, constructing stairs for leg users illustrates niche construction for locomotion, while ramps and wheelchairs serve similar purposes for people with impairments; even those deemed healthy rely on tools like clothing and shelter.^30^

What is *unfair* is that society overly prioritizes altering the environment to meet the needs of typical individuals while marginalizing those of atypical individuals. Significant advancements have been made in developing technologies to unlock the biological limitations of typical people, but comparable efforts have *not been proportionately* extended to support atypical individuals. And those using tools to aid physical functions often face bias and stigma, further deepening their disadvantages.^31^ Therefore, “society has caused inequality” encompasses not only “society doing what it should not have done,” such as fostering discriminatory attitudes and creating barriers for atypical individuals, but also “society failing to do what it should have done,” such as not fairly providing tools for atypical individuals to overcome biological limitations or ensuring non‐discrimination against their users. Accordingly, improving tool use is ethically crucial for accommodating biological diversity.

Furthermore, this social model does not inherently exclude medical interventions. A common concern with some versions of the social model advocating social changes is that they may overlook bodily experiences and the role of medical interventions in improving disabled individuals’ lives.^32^ However, a social model emphasizing societal responsibility for ensuring the proper interaction between biology and the environment can avoid this issue. If the goal is to improve the interaction, there is no good reason to exclude medical interventions that help achieve this.

In summary, the ability of people with atypical traits to function well with appropriate tools, medical support, and inclusive social attitudes, combined with the principle of fairness, supports the conclusion that society must provide comprehensive arrangements to accommodate atypical people. Simply put, “*ability” and “fairness*” underpin this claim for goal‐oriented solutions to inequality.

### 4.2 The social model of GI and ectogenesis

I apply the rationale of the above social model to GI. In this framework, socially constructed inequality arises when society fails to accommodate individuals with certain biological traits, contributing to systemic and institutional inequality. To address this, society must provide support for effective interaction with the environment. The reasoning is as follows: (1) various biological types can function well with proper social arrangements; (2) fairness requires equal consideration of different biological needs; and (3) since the goal is “good interaction,” the means are not limited to altering biological traits, tool use, or social norms.

Accordingly, a social model of GI can be viewed as society's failure to accommodate female reproductive features, necessitating comprehensive remedies. The reasoning is as follows: (1) people with female reproductive traits can interact well with their environment under proper social arrangements; (2) however, society has overwhelmingly structured around male reproductive characteristics, and thus, restoring fairness requires more efforts to improve the interaction between women's reproduction and the environment; and (3) relevant societal adjustments should be goal‐oriented, requiring comprehensive methods that do not inherently exclude ectogenesis as a significant component.

There is no reason to believe that individuals with female reproductive traits, under proper arrangements, cannot interact effectively with their environment. Good interaction can roughly mean achieving lives comparable to those with male reproductive traits. This highlights the need to ensure that RDS do not disadvantage individuals. What arrangements can achieve this? To address this, I focus on examining historical unfair arrangements to understand what the correct arrangements should entail and the role that ectogenesis might play.

#### 4.2.1 The unfairness

Society is obliged to equally address the needs of individuals with different biological traits. Historically, however, patriarchal societies have operated based on male reproductive traits, disadvantaging women in many aspects. To explore this, it is necessary to present the fundamental RDS, which I classify into two main aspects:

1. Men are not gestators; they do not experience the harm, risks, and significant time and energy investment in gestation. In contrast, women, as gestators, bear all these reproductive burdens.
2. The female body contributes far more essential biological elements to human reproduction than the male body. From conception to birth, the entire process usually takes place in the female body, meaning that women provide the gestational environment, labor, and the vital egg, while men only supply sperm. Even the egg is more crucial for embryonic development than the sperm, as it contains mitochondrial genes and other cytoplasmic structures absent in the sperm.^33^ Using cooking as an analogy: a woman provides all the tools, labor, and most ingredients, while a man provides less than half. That said, to achieve the same reproductive goal, men rely much more on women's reproductive biology than women rely on men's. As Mary O'Brien suggests, male reproduction is somewhat alienated‐being biologically unnecessary after delivering their “seed.”^34^

Simply put, female reproduction involves higher participation and contribution but comes with pain and suffering, whereas male reproduction is alienated but free from gestational harm. A just society should recognize women's reproductive contributions, strive to eliminate reproductive burden, and empower women to achieve other life goals without being disadvantaged by reproductive investment.

Nevertheless, societies have operated in the opposite direction. First, patriarchal structures prioritize alleviating male reproductive alienation at the expense of women's autonomy. Force, laws, and institutional forms like marriage, which physically isolate specific women from other men, might be conventional ways to reduce male alienation from procreation and their uncertainty of paternity.^35^ In such contexts, women's wombs were seen as “gestational carriers,” with men's preference to replace “worn‐out wombs” rather than prioritize women's health.^36^ Even new assisted reproductive technologies (ARTs) might have become complicit in this tendency. Authors like Gene Corea argue that many women using ARTs are still catering to male reproductive desires, driven by invisible emotional coercion from social norms.^37^ However, this tendency shows that coercion, not the development of ARTs, is the problem, nor is fulfilling male procreative needs inherently wrong. While men require more external elements to achieve reproductive goals than women, this is a difference, not a deficiency, in the social model. The real error lies in society's bias toward serving male reproductive traits while compromising women's reproductive autonomy.

Second, the unfairness is further manifested in the normalization and disregard of health losses that women suffer from gestation. For instance, anesthesia for childbirth once faced strong opposition for contradicting biblical teachings that saw sorrow in childbirth as a natural obligation for women.^38^ Even in today's United Kingdom, those who seek elective cesareans or epidurals for pain relief still face significant barriers.^39^ As Smajor and Räsänen note, health professionals often implement extensive and invasive medical interventions to manage fetal risks, and yet, they frequently deny or delay necessary medical support for pregnant individuals by normalizing their conditions.^40^ Contrary to seeming contradictory, the overmedicalization and undermedicalization are unified in their harm to pregnant individuals. Globally, pregnancy complications are rising due to factors like advanced maternal age, and yet, safe treatments remain scarce, hindered by systemic sexism and paternalism.^41^ Over one‐third of women worldwide face long‐term postpartum complications, and yet, these issues are largely ignored.^42^ So far, most breakthrough reproductive technologies focus on enabling physical pregnancy rather than gestational alternatives—a trend that is hardly coincidental but a consequence of societal reluctance to embrace non‐female‐exclusive gestation that could disrupt traditional family structures.^43^

Third, women have been historically confined to primary childcare, restricting their pursuit of other life goals and exacerbating this unfairness. The pressure to be a “perfect mother” still strains women's career ambitions, even in contemporary liberal states.^44^ Binding women to reproductive roles, whether through restricted access to public spheres or social norms, does not reflect their importance in human reproduction but quite the opposite. Perhaps a non‐reproductive analogy can better illustrate this injustice, given that the topic of pregnancy often triggers emotional reactions that cloud logical reasoning.

Suppose person A has an ability that person B lacks, and this ability can achieve a shared important goal. However, using this ability consumes a significant amount of A's time, energy, and health, and A has other goals like B's. For fairness, A's sacrifices should be recognized and compensated. Restricting A to the role of exercising this ability further limits their pursuit of other life goals. Therefore, a fair society should aim to reduce A's investment in achieving the common goal while providing *extra* support for A in pursuing their other life goals. That said, shared housework, child‐rearing, and equal pay—frequently emphasized by advocates of the social model—are insufficient to achieve true fairness; additional support is necessary.

In general, the patriarchal response to RDS has been to assume that only male reproduction matters, prioritizing the fulfillment of male procreative desires—especially the certainty of paternity—at the expense of women's reproductive autonomy. Instead of reducing women's reproductive burden, society has disciplined them to embrace these burdens and confined them to primary childcare roles. In this way, women and their wombs together become subjects to male procreative desires.

#### 4.2.2 The goal‐directed solutions

The moral compass derived from the social model of GI suggests that society is obligated to invest *more* effort into accommodating women's reproductive characteristics for fairness. Based on the previous discussion of how relevant unfairness operates, restoring fairness generally requires society to eliminate practices that harm women's *autonomy* to satisfy male reproductive desires, reduce women's reproductive *burden*, and empower women to pursue *life goals* beyond reproduction.

Theoretically, under the right circumstances, ectogenesis may become a crucial tool in achieving these goals. Eliminating physical gestational burdens, especially harm and risk, is the most certain benefit of ectogenesis. For this goal, ectogenesis serves as a fundamental extension of relevant medical care. Regarding autonomy, elective ectogenesis could effectively address involuntary male childlessness.^45^ If the fundamental motive behind gender oppression is male envy of pregnancy capability,^46^ ectogenesis could provide a significant reason for men to relinquish their oppressive motivations. Also, compared to existing ARTs, which usually require hormone‐induced super‐ovulation and procedures to harvest oocytes and implant embryos^47^—ectogenesis, especially when combined with in vitro gametogenesis to develop gametes from somatic cells,^48^ can eliminate invasive procedures on women. Not only can ectogenesis free women's health, time, and energy during pregnancy to pursue other life goals but it can also arguably weaken the ideological link between women's identity and reproduction,^49^ empowering them to embrace diverse aspirations. Thus, within the social model, ectogenesis can be a significant component in addressing GI.

However, this does not mean that ectogenesis is necessarily the most reasonable choice in this model. As previously discussed, another morally appealing option is the combination of easy and safe pregnancy with other proper arrangements. People may have good reasons to choose it, particularly because some women may highly value the specific gestational ties formed during pregnancy.^50^ Which option is better? The social model itself cannot provide a definitive answer. It focuses on ensuring good interaction between biology and the environment. If good interaction means meeting people's actual needs based on their values and preferences, then, like the biological model, the moral desirability of ectogenesis would be determined by democratic choice, making it an open question. If good interaction is based on some objective standard, then the two options should be evaluated according to that standard, which also means that the social model cannot provide the answer.

Hence, there is no need to reject ectogenesis to show that GI is socially caused—the social model can accommodate ectogenesis in principle. In other words, there might be other reasons to oppose ectogenesis but “society has caused GI” is not a plausible one. Meanwhile, it is also difficult to directly justify ectogenesis from this model; a comprehensive evaluation of potential future scenarios within particular normative theories might be necessary, though it is beyond the scope of this paper.

## 5 RECONCILING THE BIOLOGICAL AND SOCIAL

When GI is viewed through reasonable biological and social models, its primary cause is society's improper response to female reproductive traits. This supports comprehensive solutions not limited to medical support, tool use, or social modifications, providing an initial defense for ectogenesis. To recap:

1. Both models are inherently interactionist. Claiming that RDS has caused GI places pregnancy at the beginning of the causal chain. When interpreted through Dworkin's theory, women are entitled to compensation for pregnancy's adverse effects. Society has failed to provide this compensation, instead addressing male reproductive alienation at the expense of women's autonomy and interests, reinforcing male dominance, and deeply contributing to systemic inequality. Meanwhile, claiming that SS has caused GI still largely links with biological factors—society's disregard and marginalization of female reproductive traits are crucial to gender oppression. Acknowledging that women's struggles in patriarchal societies predominantly revolve around motherhood, marriage, and gender bias—deeply connected to pregnancy—we cannot deny the significant role of RDS in GI. Still, when society fails to fairly accommodate RDS, systemic inequality arises.
2. Both reject a hierarchy between RDS and assign accountability for redressing unfairness to society. In a reasonable biological model, the debilitation and risks of female reproduction, and the alienation of male reproduction, can be seen as two forms of natural inequality or unchosen misfortunes. This model calls for society to collectively bear the former misfortune while avoiding the sharing of the latter in ways that harm women's autonomy. These two “natural inequalities” are seen as two “differences” in the social model. A reasonable social model similarly calls for equal efforts to accommodate them, preventing society from solely focusing on addressing male alienation. In both cases, moral accountability lies with society, not biology or individuals. Failure to fairly compensate or accommodate RDS creates GI.
3. Both advocate for comprehensive solutions to GI, offering only a preliminary defense of ectogenesis. Enhancing interaction itself is the correct path to equality, not limited to targeting biology or society. Practices—whether bypassing bodily pregnancy through ectogenesis or adjusting societal norms—are responses to inequality. Ectogenesis can be seen both as compensation for women's reproductive burden and as societal action to increase efforts to accommodate female reproductive biology. It is biological because it bypasses physical pregnancy; it is social because it reflects society's self‐correction. Still, neither framework can directly dictate that strategies including ectogenesis are necessarily superior. This is precisely the core impasse in the relevant feminist debate: should we pursue a path without ectogenesis, but with enhanced medical support and empowerment for women to achieve goals beyond reproduction, or should we seek a path that integrates ectogenesis to spark a series of transformative changes for equality? Which path is more feasible and morally preferable? The debate on the origin of GI cannot provide an answer.

Therefore, for the pro‐ectogenesis stance, the narrative that “GI is rooted in pregnancy” is not a strong crutch. The potential benefits of ectogenesis emphasized by these authors, such as improving reproductive health, reducing reproductive burden, and weakening the connection between female identity and reproduction, are important dimensions in addressing GI, without relying on that prop. Focusing on exploring how these benefits can trigger a series of transformative changes in various contexts may be a more persuasive approach. Meanwhile, the narrative that “GI is primarily social” does not justify dismissing the emancipatory potential of ectogenesis—there may be other reasons to critique ectogenesis, but this is not one of them; calls for broader programs need not rely on this assumption either. That said, weighing GI's biological and social contributors is not an enlightening start for our debate; it is better to set it aside and direct attention to the potential changes that ectogenesis may bring across scenarios.

## Footnotes

1 Firestone, S. (2015 [1970]). *The dialectic of sex*. Verso Books; Smajdor, A. (2007). The moral imperative for ectogenesis. *Cambridge Quarterly of Healthcare Ethics*, *16*(3), 336–345; Smajdor, A. (2012). In defense of ectogenesis. *Cambridge Quarterly of Healthcare Ethics*, *21*(1), 90–103; MacKay, K. (2020). The ‘tyranny of reproduction’: could ectogenesis further women's liberation *Bioethics*, *34*, 346–353; Kendal, E. (2015). *Equal opportunity and the case for state sponsored ectogenesis*. Palgrave.

2 Cavaliere, G. (2020a). Gestation, equality, and freedom: Ectogenesis as a political perspective. *Journal of Medical Ethics*, *46*(2), 76–82; Cavaliere, G. (2020b). Ectogenesis and gender‐based oppression: Resisting the ideal of assimilation. *Bioethics*, *34*, 727–734; Campo‐Engelstein, L. (2020). Reproductive Technologies are not the cure for social problems. *Journal of Medical Ethics*, *46*, 85–86; Horner, C. (2020). Imagine a world… where ectogenesis isn't needed to eliminate social and economic barriers for women. *Journal of Medical Ethics*, *46*, 83–84.

3 Dworkin, R. (2000). *Sovereign virtue: The theory and practice of equality* (pp. 73–83). Harvard University Press.

4 Amundson, R. (2000). Against normal function. *Studies in History and Philosophy of Biological and Biomedical Sciences*, *31*(1), 33–53.

5 Firestone, op. cit. note 1, pp. 175–190.

6 Ibid.

7 Smajdor, op. cit. note 1.

8 Campo‐ Engelstein, op. cit. note 2; Horner, op. cit. note 2.

9 Cavaliere, op. cit. note 2.

10 Ibid.

11 Ibid.

12 Ibid.

13 Kolk, M. (2019). Weak support for a U‐shaped pattern between societal gender equality and fertility when comparing societies across time. *Demographic Research, 40*, 27–48.

14 Horner, op. cit. note 2.

15 Dworkin, op. cit. note 3.

16 Smajdor, op. cit. note 1.

17 Smajdor (2007), op. cit. note 1.

18 Ibid:243.

19 Ibid.

20 Smajdor (2012), op. cit. note 1.

21 Dworkin, op. cit. note 3, p. 78.

22 Buchanan, A., Brock, D. W., Daniels, N., & Wikler, D. (2000). *From chance to choice: Genetics and justice*. Cambridge University Press.

23 Smajdor, op. cit. note 1.

24 Ibid.

25 Ibid.

26 Shakespeare, T. (2006). *Disability rights and wrongs* (1st ed.). Routledge.

27 Amundson, op. cit. note 4.

28 Ibid.

29 Odling‐Smee, F. J., Laland, K. N., & Feldman, M. W. (2003). *Niche construction: The neglected process in evolution*. Princeton University Press.

30 Lewens, T. (2015). *The biological foundations of bioethics* (p. 179). Oxford University Press.

31 Amundson, op. cit. note 4.

32 Manago, B., Davis, J. L., & Goar, C. (2017). Discourse in action: Parents'use of medical and social models to resist disability stigma. *Social Science & Medicine, 184*, 169–177.

33 Lewens, T. (2021). The fragility of origin essentialism: Where mitochondrial ‘replacement’ meets the non‐identity problem. *Bioethics*, *35*(7), 615–622.

34 O'Brien, M. (1981). *The politics of reproduction*. Routledge & Kegan Paul.

35 Ibid.

36 Romanis, E. C., Begović, D., Brazier, M. R., & Mullock, A. K. (2021). Reviewing the womb. *Journal of Medical Ethics, 47*(11), 820–829.

37 Corea, G. (1988). *The mother machine: Reproductive technologies from artificial insemination to artificial wombs*. The Women's Press.

38 Pence, G. (2006). What's so good about natural motherhood? (In praise of unnatural gestation). In S. Gelfand & J. R. Shook (Eds.), *Ectogenesis: Artificial womb technology and the future of human reproduction* (pp. 77–88). Rodopi.

39 Nelson, A. (2024). Medical authority and expectations of conformity: Crystallising a key barrier to person‐centred care during labour and childbirth. *Journal of Medical Ethic*, *51*(2), 107–110.

40 Smajdor, A., & Räsänen, J. (2024). Is pregnancy a disease? A normative approach. *Journal of Medical Ethics*, *51*(1), 37–44.

41 Stock, S. J., & Aiken, C. E. (2023). Barriers to progress in pregnancy research: How can we break through? *Science*, *380*(6641), 150–153.

42 Vogel, J. P., Jung, J., Lavin, T., Simpson, G., Kluwgant, D., Abalos, E., Diaz, V., Downe, S., Filippi, V., Gallos, I., Galadanci, H., Katageri, G., Homer, C. S. E., Hofmeyr, G. J., Liabsuetrakul, T., Morhason‐Bello, I. O., Osoti, A., Souza, J. P., Thakar, R., … Oladapo, O. T. (2024). Neglected medium‐term and long‐term consequences of labour and childbirth: A systematic analysis of the burden, recommended practices, and a way forward. *The Lancet Global Health*, *12*(2), e317–e330.

43 Firestone, op. cit. note 1; Bidoli, A. (2024). More than an idea: Why ectogestation should become a concrete option. *Journal of Medical Ethics*. Advance online publication. https://doi.org/10.1136/jme-2023-109716

44 Meeussen, L., & Laar, C. (2018). Feeling pressure to be a perfect mother relates to parental burnout and career ambitions. *Frontiers in Psychology*, *9*, 2113.

45 Räsänen, J. (2024). Ectogestation for men: Why aren't we talking about it? *Journal of Medical Ethics*. Advance online publication. https://doi.org/10.1136/jme-2024-110128

46 O'Brien, op. cit. note 34.

47 Inhorn, M. C., & Birenbaum‐Carmeli, D. (2008). Assisted reproductive technologies and culture change. *Annual Review of Anthropology, 37*, 177–196.

48 Pennings, G. (2023). Why we need stem‐cell derived gametes. *Reproductive BioMedicine Online, 47*(5), 103322.

49 MacKay, op. cit. note 1.

50 Kennedy, S. (2024). Ectogenesis and the value of gestational ties. *Bioethics*, *38*(7), 643–649.

